# Local vs. Global Blood Flow Modulation in Artificial Microvascular Networks: Effects on Red Blood Cell Distribution and Partitioning

**DOI:** 10.3389/fphys.2020.566273

**Published:** 2020-09-25

**Authors:** Alberto Mantegazza, Matteo Ungari, Francesco Clavica, Dominik Obrist

**Affiliations:** ^1^ARTORG Center for Biomedical Engineering Research, University of Bern, Bern, Switzerland; ^2^Integrated Actuators Laboratory, École Polytechnique Fédérale de Lausanne, Neuchâtel, Switzerland

**Keywords:** cerebral blood flow, red blood cells, phase separation, neurovascular coupling, pericytes

## Abstract

Our understanding of cerebral blood flow (CBF) regulation during functional activation is still limited. Alongside with the accepted role of smooth muscle cells in controlling the arteriolar diameter, a new hypothesis has been recently formulated suggesting that CBF may be modulated by capillary diameter changes mediated by pericytes. In this study, we developed *in vitro* microvascular network models featuring a valve enabling the dilation of a specific micro-channel. This allowed us to investigate the non-uniform red blood cell (RBC) partitioning at microvascular bifurcations (*phase separation*) and the hematocrit distribution at rest and for two scenarios modeling capillary and arteriolar dilation. RBC partitioning showed similar phase separation behavior during baseline and activation. Results indicated that the RBCs at diverging bifurcations generally enter the high-flow branch (*classical partitioning*). Inverse behavior (*reverse partitioning*) was observed for skewed hematocrit profiles in the parent vessel of bifurcations, especially for high RBC velocity (i.e., arteriolar activation). Moreover, results revealed that a local capillary dilation, as it may be mediated *in vivo* by pericytes, led to a localized increase of RBC flow and a heterogeneous hematocrit redistribution within the whole network. In case of a global increase of the blood flow, as it may be achieved by dilating an arteriole, a homogeneous increase of RBC flow was observed in the whole network and the RBCs were concentrated along preferential pathways. In conclusion, overall increase of RBC flow could be obtained by arteriolar and capillary dilation, but only capillary dilation was found to alter the perfusion locally and heterogeneously.

## 1. Introduction

Neurovascular coupling, also known as functional activation or hyperemia, refers to cerebral blood flow (CBF) regulation mechanisms enabling an adequate supply of oxygen and nutrients to a localized region of the cerebral capillary network in response to increased metabolic needs (Iadecola, 2017). The interplay of neurons, glia and vascular cells regulates the CBF during an increased neuronal activity by vasodilating arterioles and capillaries (Tian et al., 2010; Hall et al., 2014; Schmid et al., 2017). In case of severe pathologies like Alzheimer's disease or ischemic stroke, the neurovascular coupling is disrupted and the activated areas of the brain are no longer supplied with sufficient blood flow (Girouard and Iadecola, 2006).

It is known that vascular smooth muscle cells (VSMC) play a role in blood flow regulation by changing the diameter of pial and penetrating arterioles, thus affecting the pressure and blood flow distributions in the downstream capillary network (Lacolley et al., 2012). Many *in vivo* studies reported arteriolar dilations or constrictions in function of neuronal activity during functional hyperemia (Fernandez-Klett et al., 2010; Hall et al., 2014; Hill et al., 2015; Mishra et al., 2016).

Recently, it has been reported that capillaries are contractile and pericyte-mediated blood flow regulation mechanisms can also take place at the level of the capillary bed (Peppiatt et al., 2006; Hall et al., 2014; Fernandez-Klett and Priller, 2015; Alarcon-Martinez et al., 2019). Pericytes are able to induce capillary dilation or contraction upon chemical and electrical stimulation (Peppiatt et al., 2006). Hall et al. (2014) found that the capillary dilation upon *in vivo* stimulation is faster than arteriolar dilation. This suggests that the neural-pericyte response is faster than the arteriolar signaling path which could play an important role in local blood flow regulation. In contrast, Hill et al. (2015) claim that even though pericytes are contractile *in vivo*, the cerebral blood flow is not mediated by capillaries but rather by VSMCs at the arteriolar level. A consensus on the spatio-temporal response of arterioles and capillary is yet to be achieved.

Many experimental studies (Kleinfeld et al., 1998; Schulte et al., 2003; Stefanovic et al., 2008; Gutiérrez-Jiménez et al., 2016) observed a heterogeneous blood perfusion in capillary networks *in vivo*. This heterogeneity is expressed by differences in the red blood cell (RBC) distribution across the capillary network. Nonetheless, many fluid dynamic characteristics of capillary perfusion during baseline and activation remain unknown. None of the previously reported studies relied on a detailed fluid-dynamical model to investigate the effects of perfusion pressure variations, diameter changes and hematocrit distribution differences on the capillary network perfusion. Quantitative data on such variables are not always measurable *in vivo* and it is difficult to isolate the contribution of each of them for the blood flow regulation.

To understand regulation mechanisms during functional activation it is essential to study the fluid dynamics in capillary networks and take into account the RBC dynamics and partitioning at the level of microvascular bifurcations. The heterogeneity in the local blood flow distribution is a direct consequence of the non-uniform RBC separation between daughter branches of diverging bifurcations (Schmid et al., 2019). At diverging bifurcations the outflow branch with a higher flow rate usually receives a disproportionally higher RBC fraction [phenomenon known as Zweifach-Fung effect (Fung, 1973; Doyeux et al., 2011)].

If the Zweifach-Fung effect is respected, a bifurcation presents a classical partitioning. In contrast, it presents a reverse partitioning if the outflow branch with a higher blood flow rate receives a disproportionally lower RBC fraction than the low flow branch (or vice versa). The occurrence of classical and reverse partitioning was observed in numerical (Balogh and Bagchi, 2017, 2018) and experimental studies (Clavica et al., 2016; Shen et al., 2016; Mantegazza et al., 2020). Reverse partitioning was found to be more likely for high perfusion pressures (Clavica et al., 2016; Mantegazza et al., 2020), skewed hematocrit profiles in the parent vessels of diverging bifurcations (Balogh and Bagchi, 2018; Mantegazza et al., 2020) or in case of autoregulation mechanisms due to cell-cell interactions (Balogh and Bagchi, 2018). These very local phenomena have a direct impact on the larger scale of microvascular networks leading to heterogenous RBC distribution and altered flow and pressure fields (Schmid et al., 2019).

Next to experimental studies *in vivo* (Kleinfeld et al., 1998; Schulte et al., 2003; Stefanovic et al., 2008; Gutiérrez-Jiménez et al., 2016) and numerical models (Lorthois et al., 2011a,b; Lorthois and Lauwers, 2012; Schmid et al., 2017, 2019; Balogh and Bagchi, 2018), there are several *in vitro* studies investigating blood flow in artificial microvascular networks *in vitro* (e.g., Forouzan et al., 2012; Reinhart et al., 2017; Fenech et al., 2019). However, to the best of our knowledge, *in vitro* experiments modeling different mechanisms of blood flow modulation during hyperemia are limited. In the present study, we designed and fabricated a microfluidic device comprising a complex yet idealized honeycomb network of micro-channels characterized by channel sizes found *in vivo* for capillaries (channel width ≤ 9.6 μ*m*). The micro-channel network featured a side pressure chamber acting as a pneumatic valve which allowed to modify locally the cross-section of a specific micro-channel in the network. Our goal was to investigate the RBC distribution and partitioning in the micro-channel network for different conditions modeling rest and hyperemia. To this end, a baseline condition was compared to two different states of activation. First, a local increase of blood flow was obtained by a local channel dilation as it may be achieved by a pericyte activation. Second, global blood flow modulation was obtained by increasing the perfusion pressure between inlet and outlet of the network as it may be obtained by a VSMC-mediated arteriolar dilation. Biological mechanisms driving or affecting the blood regulation (e.g., neurovascular signaling or cellular stimuli) have not been taken into consideration, but a fluid dynamic perspective has been adopted exclusively.

The scope of this work is to provide solid quantitative data on the blood flow and RBC distribution at the level of capillary networks during baseline and activation and to relate this data to phenomena at the vessel/bifurcation level (e.g., the Zweifach-Fung effect and RBC phase separation). We will contribute to answering the following questions: (I) which vessels can lead to the local/global modulation of blood flow in capillary networks; (II) how does the RBC flow change when a local/global blood flow modulation is imposed; (III) how do local phenomena at the level of single vessels and bifurcations relate to the hemodynamics at the network scale.

## 2. Materials and Methods

### 2.1. Microdevice Fabrication

The geometry of the microfluidic devices was designed using DraftSight (Dassault Systèmes, Vélizy-Villacoublay, France) and transferred onto a chrome photomask (JD Photodata, Hitchin, UK). Conventional soft-lithography techniques were used to fabricate a silicon master (Prolog Semicor LTD, Kiev, Ukraine). The final microfluidic devices were produced via replica molding and they were made of polydimethylsiloxane (PDMS, Sylgard 184, Dow Corning, Midland, MI, USA). A 12:1 weight ratio of pre-polymer and curing agent was prepared. More details on the fabrication process are reported by Mantegazza et al. (2020).

**Figure 1:**
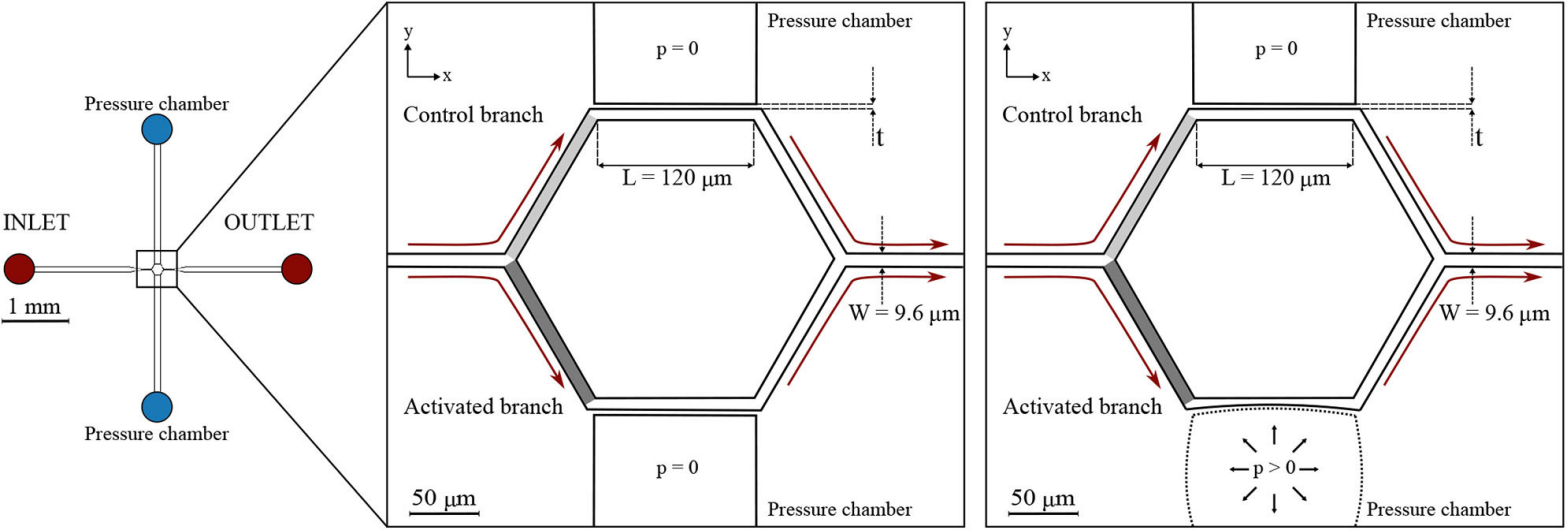
Single-mesh network. Schematic of the microfluidic device **(left)** and magnified images **(right)** with a passive (*p* = 0) or inflated (*p* > 0) pressure chamber. Regions of interest are marked in light gray and dark gray for the control branch and the activated branch, respectively. The device had a single inlet and a single outlet and embedded a single hexagonal mesh with two pressure chambers on the sides. All micro-channels had a width *W* = 9.6 μ*m*, height *H* = 8 μ*m*, and length *L* = 120 μ*m*. Two designs with different membrane thickness were used: *t*_1_ = 5 μ*m* and *t*_2_ = 10 μ*m*. The figures indicate the coordinate system: *XY* is in the focal plane of the microscope, whereas *Z* is in the perpendicular direction.

Two microfluidic devices were designed and fabricated. They comprise several micro-channels with dimensions similar to capillaries of the cerebral microvasculature (Wiedeman, 1963; Peppiatt et al., 2006; Peyrounette et al., 2018). The micro-channel sizes were designed to match the cell-to-tube diameter ratio reported in similar studies on RBC partitioning (Barber et al., 2008). The micro-channels are arranged to form networks akin to cerebral capillary beds. For the ease of writing, we call these networks *capillary networks* in the remainder of this article. The first one (hereafter referred to as *single-mesh network*) comprises a single hexagonal mesh (loop) of micro-channels and two lateral pressure chambers acting as pneumatic valves. Long drainage micro-channels (width = 100 μ*m*, height = 8 μ*m*) were placed upstream and downstream of the capillary network at the inlet and outlet. The cross-section (width × height) of all branches of the capillary network was *W* × *H* = 9.6 × 8 μ*m* while the length *L* of the micro-channels was 120 μ*m*. The hexagonal network and the pressure chambers were separated by thin membranes. Two versions of this device with different membrane thickness were tested: *t*_1_ = 5 μ*m* and *t*_2_ = 10 μ*m* (Figure 1).

**Figure 2:**
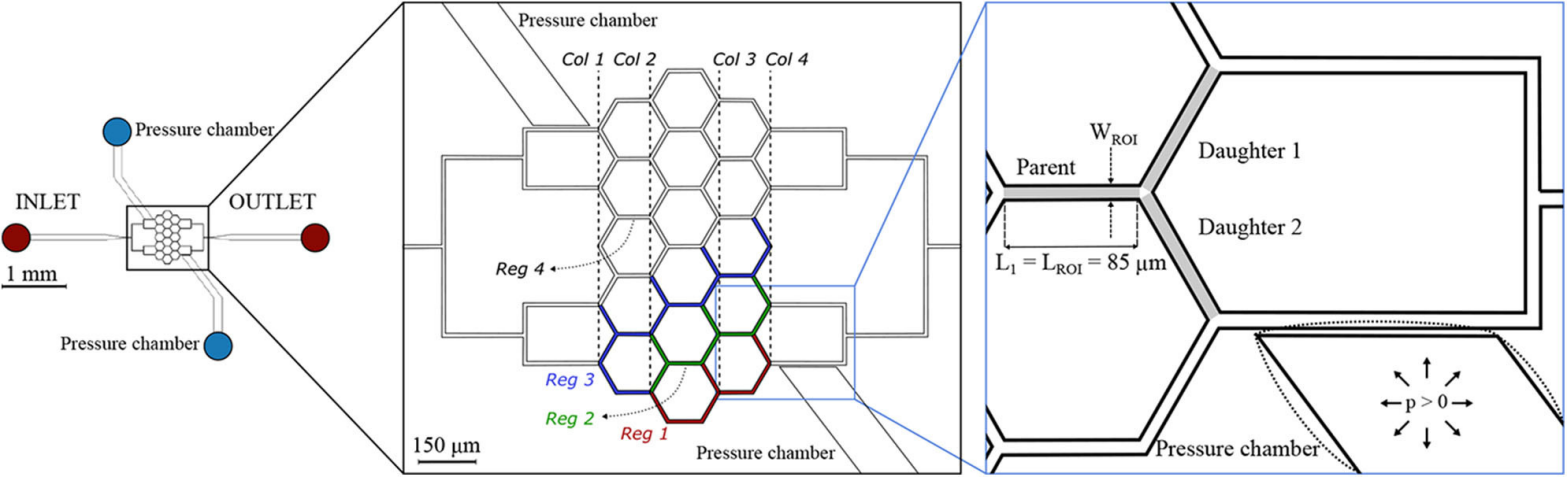
Honeycomb network. Schematic of the microfluidic device **(left)** and magnified images **(right)** with a passive (solid lines) or inflated (dotted lines) pressure chamber. The inset on the right shows an example of a diverging bifurcation. Regions of interest (ROIs) for the parent and for the daughter branches are marked in light gray. The width of the ROI is *W*_*ROI*_ = 9.6 μ*m*, while the length is *L*_*ROI*_ = 85 μ*m* for all micro-channels. The device embeds two pressure chambers which are separated from the adjacent micro-channels by thin membranes with thickness *t*_1_ = 5 μ*m*. For the interpretation of the results, four regions were defined: *Reg* 1 (red channels), *Reg* 2 (green channels), *Reg* 3 (blue channels), and *Reg* 4 (white channels). A column ordering (*Col* 1… *Col* 4) was defined to sort the diverging bifurcations.

The second microfluidic device (hereafter referred to as *honeycomb network*) features a central honeycomb network embedding 16 hexagonal elements and two T-junctions at the inlet and outlet to feed and drain the micro-channel network, respectively (Figure 2). All micro-channels had a width *W* = 9.6 μ*m*, height *H* = 8 μ*m* and length *L*_1_ = 85 μ*m*. Moreover, the microfluidic device comprises two lateral pressure chambers acting as pneumatic valves. In the present study, only the pressure chambers at the lower half of the networks were used, whereas the other pressure chambers remained passive.

It will be shown below (section 3.1), that the inflation of the pressure chamber leads to an increased cross-section of the adjacent micro-channel. This is the case, because inflation increases the height of the micro-channel more than it reduces its width.

### 2.2. Red Blood Cell Suspension

Fresh heparinized venous blood (10 *ml*) from specific pathogen-free Large White pigs was provided by the Experimental Surgery Facility (ESF, Department of Biomedical Research, University of Bern) in Bern, Switzerland. The 10 *ml* blood samples were obtained exclusively in the context of ongoing *in vivo* studies at the ESF under the animal license BE 37/19 delivered by the veterinary authorities of the Canton of Bern. The samples were centrifuged (10 *min* at 1, 800 × *g*) to separate RBCs from the other blood components and 0.5 *ml* of RBCs were collected. The RBCs were washed in a solution containing Bovine Serum Albumin (BSA) (Sigma-Aldrich, St Louis, MO, USA) dissolved at 1% in Phosphate-Buffered Saline (PBS) (Sigma-Aldrich, St Louis, MO, USA) to reduce echinocytosis (Reinhart et al., 2015). After washing, a second step of centrifugation (5 *min* at 2, 500 × *g*) was performed. A suspending medium (Roman et al., 2016) (hereafter referred to as *plasma*) was prepared by mixing 65% Glucose-Albumin-Sodium-Phosphate (GASP) buffer with 35% of stock solution (90% Optiprep (Sigma-Aldrich, St Louis, MO, USA) + 10% GASP 10 times concentrated) to match the density of the RBCs and avoid sedimentation. The final medium had a density of 1, 090 *kg*/*m*^3^ and a viscosity of 1.96 × 10^−3^ *Pa*·*s* at 20°*C*, which is in the physiological range of plasma (Fung, 1973).

RBCs were diluted in the medium at different volume fractions to produce three suspensions at 5, 10, and 25% hematocrit. We chose a feeding hematocrit in the range *H*_*f*_ = 5 − 25% to reproduce physiological values observed in the microcirculation (Pries et al., 1986; Hudetz, 1997). The feeding hematocrit used for each specific experiment has been reported in Table 1. Thanks to centrifugation steps, only RBCs were present in the final suspensions while all other cellular components were reduced. Experiments were carried out at ambient temperature (*T* = 20°*C*) within 12 h after the blood collection to preserve the healthy state of the RBCs during the experiments.

**Table 1:** The table shows which device, perfusion pressure (Δ*P*), pressure in the chamber (*p*), and feeding hematocrit (*H*_*f*_) were defined for each experiment reported in this chapter.

**Experiment**	**Device**	**Δ*P* [*mbar*]**	***p* [*bar*]**	***H*_*f*_ [*%*]**
A: Characterization	Single-mesh network (*t*_1_, *t*_2_)	0.5	0, 1, 2.25	5, 25
B: Phase separation	Single-mesh network (*t*_1_)	0.5, 1.5, 3.4	2.25	5
C: Pericyte activation	Honeycomb network	9.8	0, 2.25	10
D: Arteriolar activation	Honeycomb network	9.8, 19.6	0	10

### 2.3. 3D Characterization of Micro-Channel Geometry

Confocal fluorescence microscopy was used to evaluate the deformation of the microfluidic devices upon the inflation of the pressure chamber. Cross-sectional images in three mutually perpendicular planes aligned with the micro-channel network were acquired to perform a three-dimensional characterization of the single-mesh network. To record fluorescence images, the microfluidic device was placed on the stage of an inverted microscope (Eclipse Ti-E, Nikon, Japan) and filled with a solution of Rhodamine-B (Sigma-Aldrich, St Louis, MO, USA) and deionized water. A concentration of 0.1 *mg* of Rhodamine-B dissolved in 50 *mL* of water was used. The solution was filtered before use to remove undissolved particles of rhodamine. A green laser light with a wavelength λ_*ex*_ = 543 *nm* was used as exciting light source. Stacks of images with a lateral resolution of 0.33 μ*m*/*pixel* were recorded along the *z* direction with a Z-slice spacing Δ*z* = 0.1 μ*m* before and after the inflation of the pressure chamber (*p* = 2.25 *bar*, see section 3.1). 96 slices were acquired in total to scan the full height of the microfluidic device. Image post-processing was carried out using the open-source platform Fiji (Schindelin et al., 2012).

### 2.4. Experimental Protocol

The microfluidic devices were degassed and pre-filled with a solution of PBS and BSA (Clavica et al., 2016) before the experiment. The blood suspension at the desired hematocrit was placed in a 10 *ml* centrifuge tube, mounted on a vertical linear-motion stage and connected to the inlet of the microdevices. The hydrostatic pressure difference between the fluid level in the reservoir and the device outlet (i.e., perfusion pressure) was used to drive the blood flow. Perfusion pressures (Δ*P*) used to perform the experiments are reported in Table 1. These pressures were selected to generate a flow through the network with RBC velocities on the order of 0.5 *mm*/*s* which is a velocity found *in vivo* for capillary networks at rest (Schulte et al., 2003; Stefanovic et al., 2008; Schmid et al., 2019). The highest perfusion pressure (Δ*P* = 19.6 *mbar*) increased the velocities up to 1.2 *mm*/*s* which models increased CBF due to functional stimulation (Kleinfeld et al., 1998; Schulte et al., 2003). The change of the fluid level in the reservoir during the course of an experiment was negligible, therefore the pressure difference across the microfluidic device remained constant preventing flow rate variations. A syringe pump was connected to one of the pressure chambers and filled with deionized water and a positive hydrostatic pressure was applied to inflate the pressure chamber and deform the micro-channels (Figures 1, 2). The pressures (*p*) imposed with the syringe pump are reported in Table 1 for the experiments that required the inflation of the pressure chamber. The integrity of the pressure chambers and the absence of leakages were verified by measuring a constant pressure in the syringe pump for the whole duration of the experiments. The second pressure chamber was unused and left at atmospheric pressure without fluid in it. The microdevices were placed on the stage of an inverted microscope (Eclipse Ti-E, Nikon, Japan) with a 10× air objective (lateral resolution 0.65 μ*m*/*pixel* and Numerical Aperture = 0.45). The samples were illuminated by white light coming from a LED lamp and a region of interest of 512 × 512 *pixels* was considered to record images. For each experiment, the hydrostatic pressure difference was set first. Once a steady-state condition was reached, videos of 25 s were recorded at 395 *frames*/*second* using a high-speed camera (ORCA-flash 4.0, Hamamatsu, Japan) with (*activated* case) and without (*control* case) the inflation of the pressure chamber.

### 2.5. Image Analysis and Metrics

Custom-written Matlab (Mathworks, Natick, MA, USA) scripts were used to import and segment the recorded image sequences. The workflow comprised four main steps: correction for background illumination differences, background subtraction, noise removal and binarization (from grayscale to black and white images). The resulting frames were analyzed with the open-source software PTVlab (Brevis et al., 2011) for Particle-Tracking Velocimetry (PTV). The PTV algorithm provides the coordinates and velocity vector for all RBCs identified in a Region Of Interest (ROI) for each frame of the image sequence. This information was further processed to derive the following quantities for each frame: average RBC velocity [*mm*/*s*] and RBC number. The average RBC velocity is the arithmetic mean of the velocities of all RBCs found in the ROI for a given frame. For the single-mesh network two ROIs were defined: one for the control branch (light gray box in Figure 1) and one for the activated branch (dark gray box in Figure 1). For all diverging bifurcations present in the honeycomb network a ROI for the parent vessel and one ROI for each daughter branch were defined (Figure 2). The resulting time series of RBC velocity and number were smoothed with a second-order Savitzky-Golay filter (Mathworks, Natick, MA, USA), using a 199 point window corresponding to intervals of 0.51 s. These data were then used to compute tube hematocrit, RBC flux and blood flow rate for each ROI (sample size *n* = 9, 875). The validation of the PTV algorithm for the RBC tracking was previously performed and reported elsewhere (Mantegazza et al., 2020).

The measurement of tube hematocrit (*H*_*t*_, i.e., the ratio of the total RBC volume to channel volume), RBC flux and blood flow rate relied on the mathematical description by Mantegazza et al. (2020) which is summarized in the following.

For each frame of the image sequence the tube hematocrit was calculated as

(1) $$\begin{array}{l} H_{t}=\frac{N_{rbc}\times MCV_{rbc}}{V_{channel}}, \end{array}$$

where *N*_*rbc*_ is the number of RBCs in the ROI identified by the particle-tracking algorithm, *V*_*channel*_ is the volume of the micro-channel segment in the ROI (*V*_*channel*_ = *L*_*ROI*_ × *W*_*ROI*_ × *H*) and *MCV*_*rbc*_ is the RBC mean corpuscular volume. $MCV_{rbc}=56\;\mu m^{3}$ is the average volume of a single porcine RBC (Amin and Sirs, 1985). Temporal averaging was performed to compute the mean hematocrit.

The PTV software allowed to compute the velocity vector **U_rbc_** = [*U*_*rbc,x*_, *U*_*rbc,y*_] in the plane *XY* for all RBCs identified in a specific ROI. Assuming that *U*_*rbc*_ is the mean of the velocity magnitude of all RBCs identified in the ROI (*width* = *W*_*ROI*_, *length* = *L*_*ROI*_, *height* = *H*), the RBC flux *Q*_*rbc*_ and the total blood flow rate *Q*_*blood*_ were computed as

(2) $$\begin{array}{l} Q_{rbc}=U_{rbc}\times H_{t}\times W_{ROI}\times H=U_{rbc}\times \frac{N_{rbc}\times MCV_{rbc}}{L_{ROI}}, \end{array}$$

(3) $$\begin{array}{l} Q_{blood}=\chi \times U_{rbc}\times W_{ROI}\times H \end{array}$$

where χ is a coefficient accounting for the velocity difference between plasma and red cells. Due to the Fåhraeus effect (Fåhraeus, 1929), the RBC velocity is generally higher than the plasma velocity. Similarly to Sherwood et al. (2014), we assumed χ = 1 implying that the mean total blood velocity is equal to the mean RBC velocity. It was demonstrated (Mantegazza et al., 2020) that this is a legitimate assumption to derive the phase separation diagram because the results are independent of the choice of χ (see Equation 4). In the experiment C and D (Table 1), we computed hematocrit, RBC velocity and blood flow relative differences to compare control conditions against pericyte or arteriolar activation. For a generic quantity X, the relative difference was calculated as [(*X*_*activated*_ − *X*_*control*_)/*X*_*control*_] × 100.

To study the phase separation at diverging bifurcations in the honeycomb network RBC flux and blood flow rate data were corrected in a manner similar to Mantegazza et al. (2020) by enforcing mass conservation at each bifurcation [correction procedure first introduced by Pries et al. (1989). The corrected flow values for the parent vessel (${\hat{Q}}^{P}$) and the daughter branches (${\hat{Q}}^{1}$ and ${\hat{Q}}^{2}$) of a generic bifurcation were used to compute the fractional blood flow (Φ^*i*^) and the fractional RBC flux (Ψ^*i*^) in the daughter branch *i* as

(4) $$\begin{array}{l} \Phi^{i}=\frac{{\hat{Q}}_{blood}^{i}}{{\hat{Q}}_{blood}^{P}},\;{\text{Ψ}}^{i}=\frac{{\hat{Q}}_{rbc}^{i}}{{\hat{Q}}_{rbc}^{P}} \end{array}$$

where *i* = 1, 2 denotes the daughter branch 1 and 2 of a generic diverging bifurcation (Figure 2).

The measured values for Φ^*i*^ and Ψ^*i*^ were fitted to the commonly used *logit* function for phase separation proposed by Pries and Secomb (2008) which is defined as

(5) $$\begin{array}{l} {\text{Ψ}}_{Pries}^{i}=A+B\times logit\left(\frac{\Phi^{i}-X_{0}}{1-2X_{0}}\right) \end{array}$$

where *logit*(*x*) = *ln*(*x*/(1 − *x*)) while *A*, *B*, and *X*_0_ are dimensionless parameters. *A* describes the asymmetry between the daughter branches, *B* denotes the sigmoidal shape of the fitting function and *X*_0_ defines the minimum blood flow fraction Φ^*i*^ below which no RBCs flow into a daughter branch. In this study, *A* = 0 because the daughter branches have the same hydraulic diameter. The numerical parameters *B* and *X*_0_ are 1.65 and 0.089 (Mantegazza et al., 2020). They were previously multiplied by a correction factor (Roman et al., 2016) $MCV_{ratio}=\sqrt[{3}]{56/91}=0.85$ to take into account the volume difference between human RBCs for which Equation (5) was defined and porcine RBCs used in these experiments.

## 3. Results

In the following, we will first show confocal fluorescence images of the single-mesh network after the inflation of one pressure chamber (section 3.1) which set a baseline for the interpretation of the microfluidic experiments. Second, results on the characterization (section 3.1.1) and phase separation (section 3.1.2) for the single-mesh network are shown. Finally, results on the hematocrit, velocity, RBC flux distributions, and phase separation are presented for the honeycomb network for two different scenarios modeling a pericyte (section 3.2.1) or arteriolar activation (section 3.2.2).

### 3.1. Single-Mesh Network

**Figure 3:**
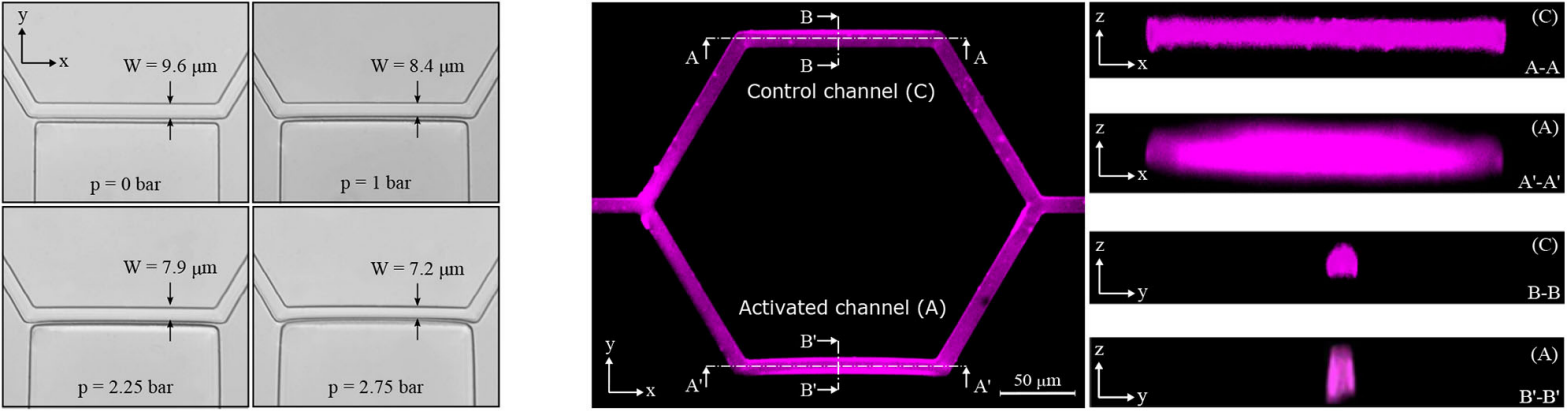
Activated channel deformation. Deformation of the activated channel in the plane *XY* upon the inflation of the pressure chamber **(left)**. Four pictures are shown starting from the baseline configuration at *p* = 0 *bar* to the maximum deformation reached at *p* = 2.75 *bar* (magnification 40×, lateral resolution 0.16 μ*m*/*pixel*). Fluorescence images for the single-mesh network upon the inflation of the pressure chamber (*p* = 2.25 *bar*) in the planes *XY*, *XZ*, and *YZ* **(right)**. Images in the *z* direction show differences between the control channel (C) and the activated channel (A).

In the single-mesh network the inflation of the pressure chamber induced a deformation of the adjacent micro-channel. Figure 3 (left) shows four microscope images illustrating a progressive deformation of the micro-channel when the pressure in the chamber is increased stepwise from 0 to 2.75 *bar*. In the *XY* plane, the nominal width of the micro-channel was *W* = 9.6 μ*m* for the control configuration (*p* = 0 *bar*). For the maximum pressure in the chamber (*p* = 2.75 *bar*) the measured width of activated micro-channel was 7.2 ± 0.1 μ*m*.

To investigate geometry changes in the planes perpendicular to the focal plane of the microscope (i.e., *XZ* and *YZ*), confocal fluorescence images were recorded. It was not possible to stain the transparent walls of the microfluidic device. Therefore, rhodamine-B was dissolved in deionized water such that we could gather a fluorescence signal from the fluid filling the microfluidic device and qualitatively reconstruct the micro-channel geometry. Figure 3 (right) shows confocal fluorescence images for the single-mesh network upon the inflation of the pressure chamber at *p* = 2.25 *bar*. We found that the inflation of the pressure chamber caused a contraction in the plane *XY* (reduced width) and a dilation in the plane *XZ* (increased height) in the adjacent micro-channel. Overall this led to an increase in the cross-sectional area of the activated channel (see Figure 1) compared to the control channel. Despite difficulties due to the diffused and scattered fluorescence signal, we were able to measure a relative cross-section increase of 39% at the point of maximum deflection (see section B′-B′ in Figure 3 on the right). Assuming an undeformed cross-section at the two extremities of the activated channel and linear increase of deflection along the length of the micro-channel, we estimated a mean cross-sectional increase of 20% in the activated channel compared to the control channel. This result is consistent to the particle-tracking data showing increased velocity in the activated branch compared to the control branch (e.g., **Figure 5** in section 3.1.2).

#### 3.1.1. Experiment A: Characterization

The characterization experiments were performed to investigate the capability of the single-mesh model to induce a blood flow variation when the pressure chamber is inflated. Two versions of the microfluidic device (membrane thickness *t*_1_ = 5 μ*m* and *t*_2_ = 10 μ*m*) were tested for different chamber pressures *p* and feeding hematocrits *H*_*f*_. To illustrate the effects of a localized dilation induced by the inflation of the pressure chamber, we report the RBC flux ratio between the activated (A) branch and the control (C) branch *q*_*A*/*C*_ = *Q*_*rbc,A*_/*Q*_*rbc,C*_ (see ROIs in Figure 1). It is important to underline that the presented data were sampled from ROIs which did not undergo any geometry change upon the inflation of the pressure chamber. Therefore, any reported velocity variation was exclusively due to the reduced hydraulic resistance induced by the cross-section increase in the downstream activated channel.

**Figure 4:**
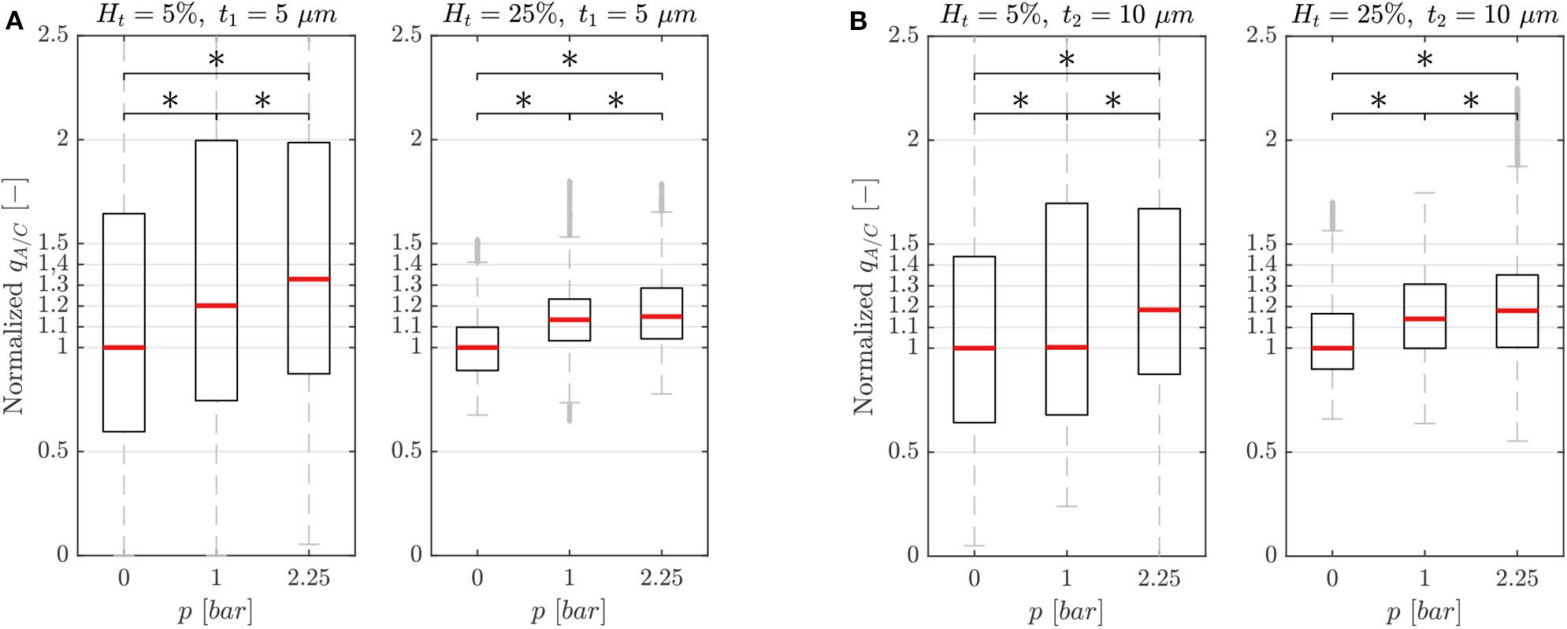
Characterization results. Characterization results for the single-mesh network with membrane thickness *t*_1_ = 5 μ*m* **(A)** and *t*_2_ = 10 μ*m* **(B)**. Data were sampled from the ROIs illustrated in Figure 1 and show the RBC flux ratio between the activated and control branch in function of the hematocrit *H*_*t*_ and pressure *p* in the pressure chamber (^*^p-value < 0.05, boxes indicate the 25th and 75th percentiles; red lines: median values; gray + are outliers). The RBC flux ratios in each subplot have been normalized with respect to the median value of the respective RBC flux ratio at *p* = 0 *bar*.

In general, the dilation of the activated channel caused an asymmetry in the RBC flux partition (Figure 4) with the activated branch receiving a higher RBC flux fraction. For both membrane thicknesses (*t*_1_ and *t*_2_) and for all experimental settings, the degree of asymmetry in the flux partition increased significantly with the pressure *p* in the chamber (*p*-value < 0.05, Wilcoxon ranksum test). For the single-mesh network with a smaller membrane thickness (*t*_1_ = 5 μ*m*, Figure 4A) at the maximum pressure *p* = 2.25 *bar*, the RBC flux ratio was *q*_*A*/*C*_ = 1.33 and 1.15 for feeding hematocrit *H*_*t*_ = 5 and 25%, respectively. Similar behavior was observed for the microfluidic device with membrane thickness *t*_2_ = 10 μ*m* (Figure 4B). The inflation of the pressure chamber at the maximum achievable pressure (*p* = 2.25 *bar*) led to a RBC flux ratio of *q*_*A*/*C*_ = 1.18 for both feeding hematocrit *H*_*t*_ = 5 and 25%. For the network with thinner membrane the RBC flux partition was dependent on the feeding hematocrit such that the degree of asymmetry in the flux partition was reduced when the feeding hematocrit was increased.

#### 3.1.2. Experiment B: Phase Separation

The phase separation experiment was performed to investigate the effect of the perfusion pressure on the RBC flux partition when the pressure chamber was inflated. Based on the results of the characterization experiment, we chose the following experimental settings which yielded the strongest variations in flux ratio: single-mesh network with membrane thickness *t*_1_ = 5 μ*m*, feeding hematocrit *H*_*f*_ = 5% and pressure *p* = 2.25 *bar*.

**Figure 5:**
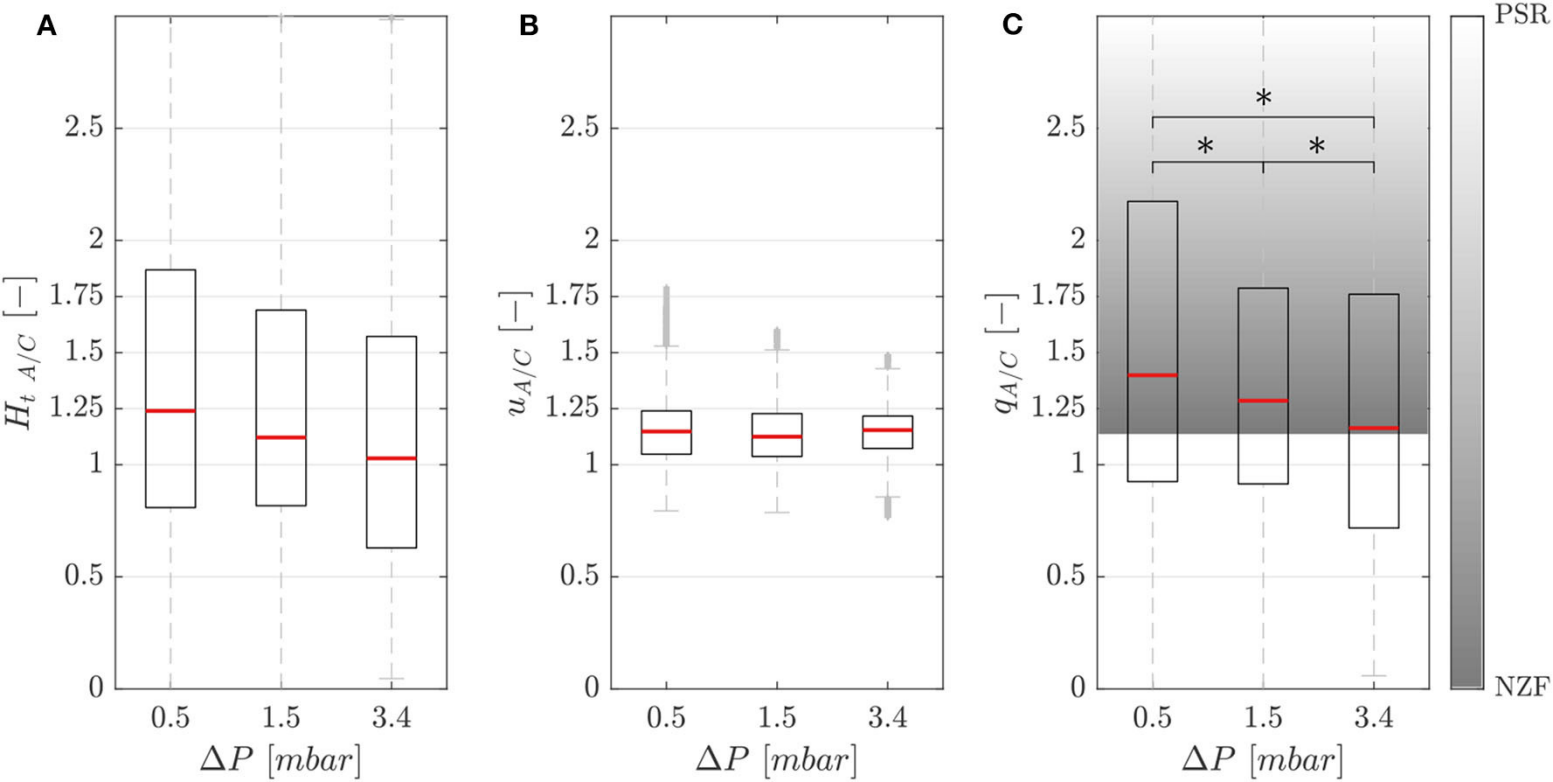
Phase separation results. Hematocrit **(A)**, velocity **(B)**, and RBC flux **(C)** ratios between the activated and control branch in function of perfusion pressure for the single-mesh network with membrane thickness *t*_1_ = 5 μ*m*, pressure in the chamber *p* = 2.25 *bar* and feeding hematocrit *H*_*f*_ = 5% (^*^p-value < 0.05). The shaded area represents the range between the no-Zweifach-Fung (NZF) and perfectly-self-regulated (PSR) conditions. Boxes indicate the 25th and 75th percentiles; red lines: median values; gray + are outliers.

Figure 5A shows the hematocrit ratio *H*_*t A*/*C*_ = *H*_*t,A*_/*H*_*t,C*_ between the activated branch and the control branch for different perfusion pressure Δ*P*. Generally, the activated branch received more RBCs than the control branch. However, a decrease of 18% was measured when passing from Δ*P*_1_ = 0.5 *mbar* to Δ*P*_3_ = 3.4 *mbar*.

Figure 5B shows that the RBC velocity in the activated branch was higher than in the control branch for all perfusion pressures. The RBC velocity ratio *u*_*A*/*C*_ = *U*_*rbc,A*_/*U*_*rbc,C*_ was 1.15, 1.13, and 1.15 for the perfusion pressures Δ*P*_1_ = 0.5 *mbar*, Δ*P*_2_ = 1.5 *mbar* and Δ*P*_3_ = 3.4 *mbar*, respectively.

Figure 5C shows the RBC flux ratio *q*_*A*/*C*_ in function of the perfusion pressure. According to Clavica et al. (2016), two extreme situations were defined to interpret the RBC phase separation results: if the RBC separation is proportional to the respective blood flow in the daughter branches, we call this a no-Zweifach-Fung (NZF) situation. If the autoregulation mechanisms based on the Zweifach-Fung effect lead to equal blood flow in the two branches, we define this configuration as perfectly-self-regulated (PSR). In accordance with the Zweifach-Fung effect, the activated branch had a higher RBC flux (because the downstream hydraulic resistance was smaller compared to the control branch) for all perfusion pressures. However, a reduction of the Zweifach-Fung effect was found with increasing perfusion pressure: the RBC flux ratio *q*_*A*/*C*_ decreased by 17% from 1.40 to 1.16 approaching the NZF limit. The Wilcoxon ranksum test was performed to compare the RBC flux ratio for different perfusion pressures. The three datasets had a *p*-value < 0.05, thus the RBC flux ratio could be considered statistically significantly lower for increasing perfusion pressure.

### 3.2. Honeycomb Network

The honeycomb network allowed to compare different scenarios connected to blood flow regulation mechanisms in capillary networks. The goal of our experiments was to investigate differences in hematocrit, velocity and RBC flux distributions and to study the RBC partitioning at diverging bifurcations when a local (section 3.2.1) or global (section 3.2.2) modulation of blood flow was implemented in the honeycomb network. Both scenarios modeling hyperemic conditions were compared to the same baseline case.

#### 3.2.1. Experiment C: Pericyte Activation

Our first study with the honeycomb network (Figure 2) compared two different scenarios: a baseline case representing control conditions (perfusion pressure Δ*P* = 9.8 *mbar*, *p* = 0 *bar* in both pressure chambers) and an activated case (perfusion pressure Δ*P* = 9.8 *mbar*, *p* = 2.25 *bar* in the bottom pressure chamber and *p* = 0 *bar* in the top chamber). The activated region was a dilated micro-channel located at the bottom right corner of the honeycomb network. The inflation of the pressure chamber served as local blood flow modulation mechanism as it may be achieved by a pericyte-induced capillary dilation *in vivo* (Hall et al., 2014). In the following, relative differences for various hemodynamic quantities between baseline and activated case are provided as median ± quartile deviation (QD).

**Figure 6:**
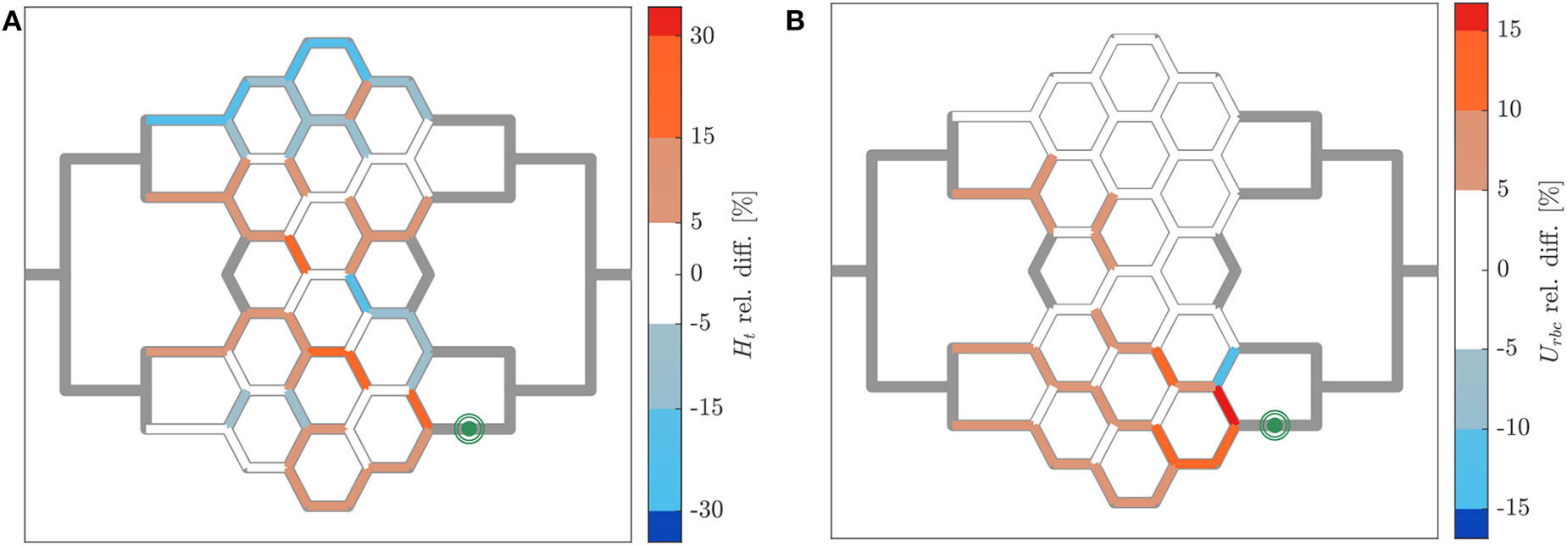
Hematocrit and RBC velocity relative difference for pericyte activation. Relative hematocrit **(A)** and RBC velocity **(B)** difference for individual micro-channels of the honeycomb network between the inflation of the pressure chamber (Δ*P* = 9.8 *mbar*, *p* = 2.25 *bar*) and the control case (Δ*P* = 9.8 *mbar*, *p* = 0 *bar*). The green symbol indicates the activated micro-channel. Flow direction is from left to right.

**Figure 7:**
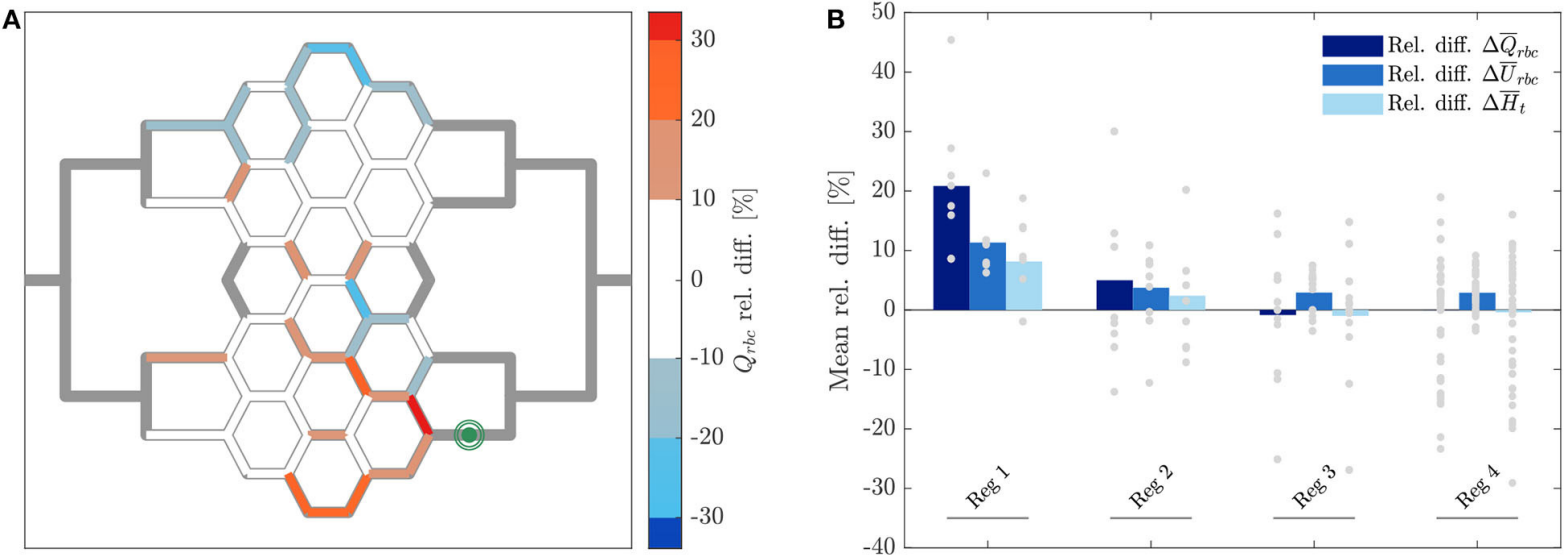
RBC flux relative difference for pericyte activation. **(A)** Relative RBC flux difference for individual micro-channels of the honeycomb network between the inflation of the pressure chamber (Δ*P* = 9.8 *mbar*, *p* = 2.25 *bar*) and the control case (Δ*P* = 9.8 *mbar*, *p* = 0 *bar*). The green symbol indicates the activated micro-channel. Flow direction is from left to right. **(B)** Mean hematocrit, RBC velocity, and RBC flux relative differences due to the activated branch different regions defined in Figure 2.

The relative hematocrit and RBC velocity differences for all individual micro-channels of the honeycomb network are shown in Figures 6A,B, respectively. We observed that the localized modulation of blood flow induced heterogeneous differences in the hematocrit distribution within the whole network. Close to the activated region (green symbols in Figure 6) a hematocrit increase up to 19% was measured (baseline: 4.91 ± 0.73, activated: 5.84 ± 0.85). On the contrary, a hematocrit decrease up to −29% (baseline: 3.47 ± 1.07, activated: 2.46 ± 1.09) was measured opposite to the activation region in the upper left corner of the honeycomb network. Differences in the RBC velocity were mostly concentrated in the surroundings of the activated region (Figure 6B). Except for two micro-channels in close vicinity to the pressure chamber which presented a velocity increase of 23% (baseline: 0.183 ± 0.008 *mm*/*s*, activated: 0.225 ± 0.007 *mm*/*s*) and a velocity decrease of −13% (baseline: 0.198 ± 0.008 *mm*/*s*, activated: 0.173 ± 0.008 *mm*/*s*), all the other channels presented a relative RBC velocity difference between −5 and 12%. The corresponding relative difference of the RBC flux is visualized in Figure 7A. The capillary dilation resulting from the inflation of the pressure chamber induced a local increase of RBC flux up to 45% (baseline: 11.86 ± 1.81 *RBC*/*s*, activated: 17.24 ± 2.48 *RBC*/*s*) in the neighborhood of the activated region. On the opposite side of the network a mix of RBC flux increase and decrease was found, whereas a flux decrease reached values up to −23% (baseline: 9.19 ± 2.77 *RBC*/*s*, activated: 7.04 ± 3.15 *RBC*/*s*).

In Figure 7B the average relative differences of hematocrit, RBC velocity and flux are presented for different sub-regions of the honeycomb network (according to Figure 2). Results were segmented in regions to analyze how the previous hemodynamic quantities change with increasing distance from the activated region. While the average relative differences of hematocrit and RBC flux increased for *Reg 1* and *Reg 2* (close to the activated micro-channel), they decreased for *Reg 3*. Farther away from the activated region (*Reg 4*), the average relative differences of hematocrit and RBC flux were zero. However, as observed in Figures 6A, 7A, changes in individual vessels are highly heterogeneous and may differ considerably from the average values. The mean RBC velocity difference increased in all regions: the mean relative difference (± *SD*) for ${\bar{U}}_{rbc}$ was higher than 10 ± 5% for *Reg 1*, while for the other regions the mean relative changes were <5% (the range of variation is shown in Figure 7B).

**Figure 8:**
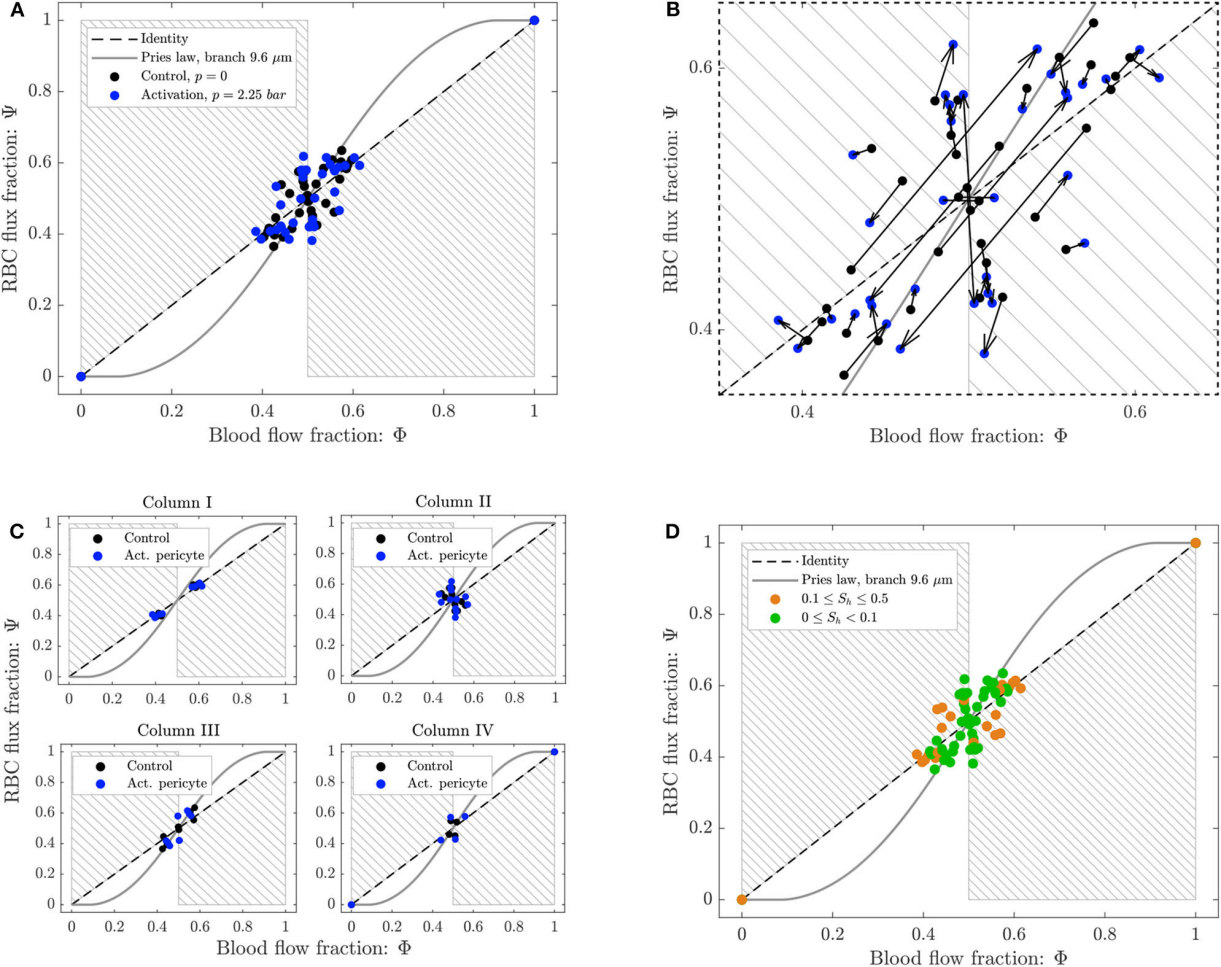
Phase separation results for pericyte activation. **(A)** Blood flow fraction vs. RBC flux fraction for the control case (black, Δ*P* = 9.8 *mbar*, *p* = 0 *bar*) and the inflation of the pressure chamber (blue, Δ*P* = 9.8 *mbar*, *p* = 2.25 *bar*). **(B)** Magnified region of **(A)**. The arrows indicate the change of the phase separation for each bifurcation. **(C)** Phase separation data shown in **(A)** grouped with respect to column ordering defined in Figure 2. **(D)** Phase separation data shown in **(A)** grouped in function of the skewness factor of the hematocrit profile in the parent vessel of each bifurcation. For all plots circles represent data from this study. The gray continuous line is the empirical law from Pries and Secomb (2008). The dashed line is the identity line representing the proportional separation of blood and RBCs (no phase separation). Data points in the gray shaded regions indicate an inversion of the Zweifach-Fung effect (Clavica et al., 2016; Shen et al., 2016).

To study the phase separation, the RBC flux fraction Ψ^*i*^ in each daughter branch of all diverging bifurcations was plotted in function of the respective blood flow fraction Φ^*i*^ (Figure 8). The results were compared with the empirical fitting of *in vivo* data from Pries and Secomb (2008).

The majority of the data lied in the range 0.4 < Φ^*i*^ < 0.6, except for two data points representing diverging bifurcations with one RBC-free daughter branch located at (0, 0) and (1, 1) in the Φ − Ψ diagram (Figure 8). Following the definition introduced by Schmid et al. (2015), we found that 89% (control) and 79% (activated) of the diverging bifurcations were well-balanced. Pries' law appears to agree well for the experimental data obtained for the control case and for the activation of the pericyte. The overall phase separation behavior of the two experiments was similar (Figure 8A). The activation of the pericyte slightly modulated the phase separation data in the Φ − Ψ diagram (Figure 8B), but for almost all bifurcations the type of partitioning (i.e., classical or reverse partitioning) remained constant.

Nonetheless, differences were found when the data points were grouped based on topological characteristics of the honeycomb network. The RBC phase separation is a local phenomenon, thus it was meaningful to follow the RBCs on their way from the inlet to the outlet of the network and, therefore, to segment the bifurcations by column number rather than considering the distance between a bifurcation and the activated region. When the bifurcations were sorted based on their respective column number (according to the notation in Figure 2), results indicated that bifurcations belonging to the same column had a similar phase separation behavior (Figure 8C). Studying the hematocrit distribution in the parent vessel of all diverging bifurcations, we found that the bifurcations could be classified in two groups depending on the shape of hematocrit profile (Supplementary Figure 1). The skewness factor (*S*_*h*_) for each hematocrit profile was computed according to Mantegazza et al. (2020). Results illustrated in Figure 8D showed that the majority of bifurcations whose parent vessels had a skewness factor *S*_*h*_ < 0.1 followed the classical partitioning (i.e., Zweifach-Fung effect) indicating that branches with higher blood flow fraction (Φ^*i*^ > 0.5) received even more RBC flux (Ψ^*i*^ > Φ^*i*^) and vice versa. In contrast, bifurcations with 0.1 ≤ *S*_*h*_ < 0.5 [orange circles in the gray shaded regions (Figure 8D)] showed a reverse partitioning (i.e., inversion of the Zweifach-Fung effect). In this case, branches with a higher blood flow fraction (Φ^*i*^ > 0.5) received less RBC flux fraction (Ψ^*i*^ < Φ^*i*^) and vice versa.

#### 3.2.2. Experiment D: Arteriolar Activation

The second set of experiments addressed arteriolar activation modeled by increasing perfusion pressure Δ*P*. Two different scenarios were investigated: a baseline case representing control conditions (perfusion pressure Δ*P* = 9.8 *mbar*, *p* = 0 *bar* in both pressure chambers) and an activated case (perfusion pressure Δ*P* = 19.6 *mbar*, *p* = 0 *bar* in both pressure chambers). An increase in the perfusion pressure acted as global modulation mechanism similar to what would occur *in vivo* due to an arteriolar dilation upstream of a capillary network. In the following, relative differences for various hemodynamic quantities between baseline and activated case are provided as median ± quartile deviation (QD).

**Figure 9:**
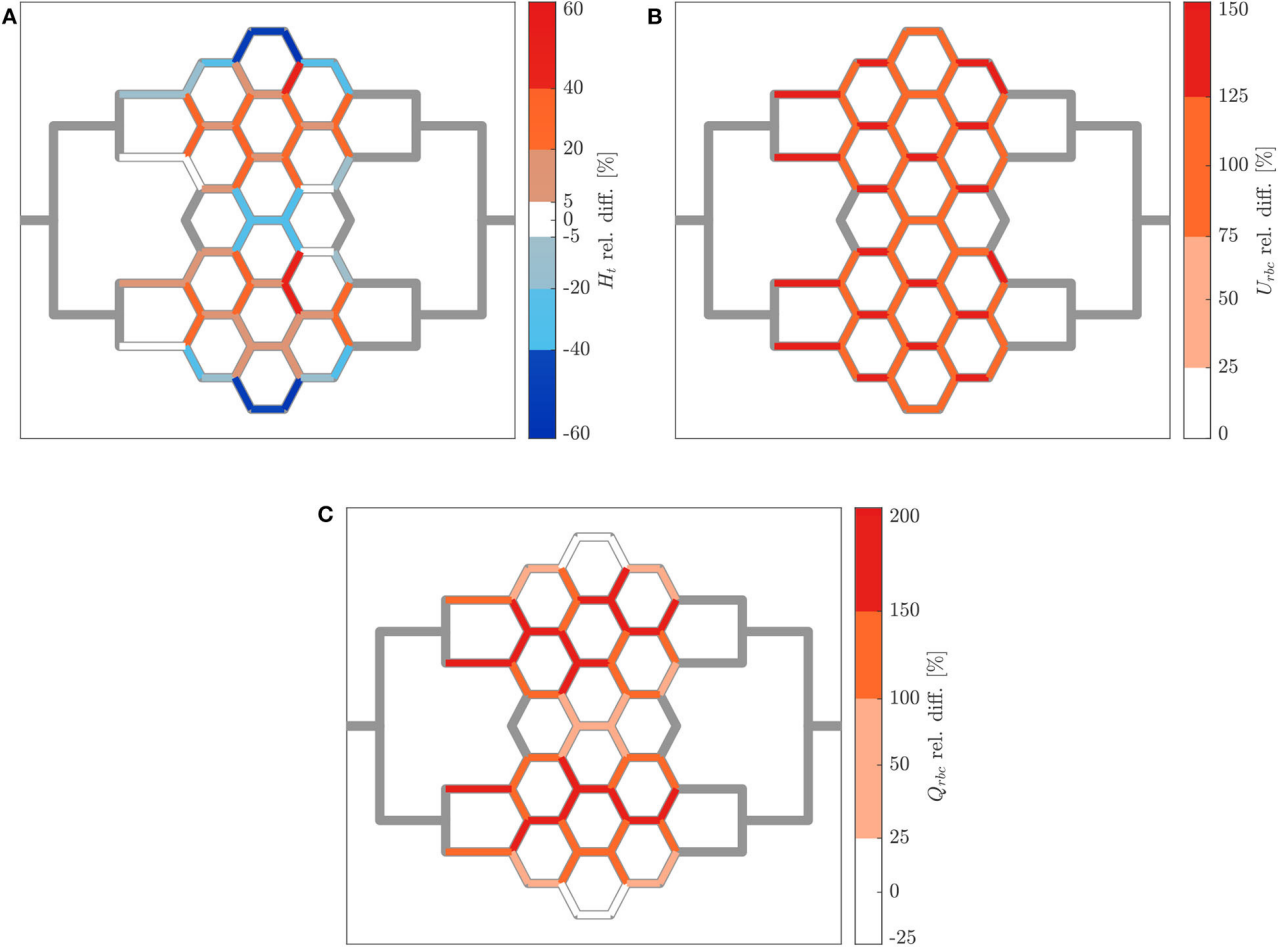
Hematocrit, RBC velocity and flux relative difference for arteriolar activation. Relative hematocrit **(A)**, RBC velocity **(B)**, and flux **(C)** difference for individual micro-channels of the honeycomb network between the activation of an upstream arteriole modeled by increasing the pressure difference between inlet and outlet of the honeycomb network (Δ*P* = 19.6 *mbar*, *p* = 0 *bar*) and the control case (Δ*P* = 9.8 *mbar*, *p* = 0 *bar*). Flow direction is from left to right.

The relative hematocrit difference for all individual channels of the network is shown in Figure 9A. The change in RBC distribution was heterogeneous. For the activated case, a RBC accumulation was found in most of microchannels of *Reg 2* and *Reg 3* (and in their corresponding regions in the upper half of the network) and a median hematocrit increase of 22 ± 9% was measured. A RBC reduction was found in the micro-channels along the horizontal axis of symmetry of the network and in the most peripheral micro-channels at the top and bottom of the network. The median hematocrit decrease for these micro-channels was −28 ± 12%.

Figure 9B shows how the increased perfusion pressure affected uniformly the RBC velocity in the whole network for the activated case. The RBC velocity increased in all micro-channels and the median of the RBC velocity relative difference was 109 ± 16%. This result is consistent with the perfusion pressure imposed for this experiment which was 2-fold the perfusion pressure for the control case. The corresponding relative difference of the RBC flux is shown in Figure 9C. Preferential pathways were identified within *Reg 2* and *Reg 3* (and in their corresponding regions in the upper half of the network). In general, a global RBC flux increase was present in the network with a median relative difference of 140 ± 42%. The only exception were six micro-channels at the periphery of the network which presented a median RBC flux decrease of −17 ± 7% as a result of the hematocrit decrease shown in Figure 9A.

**Figure 10:**
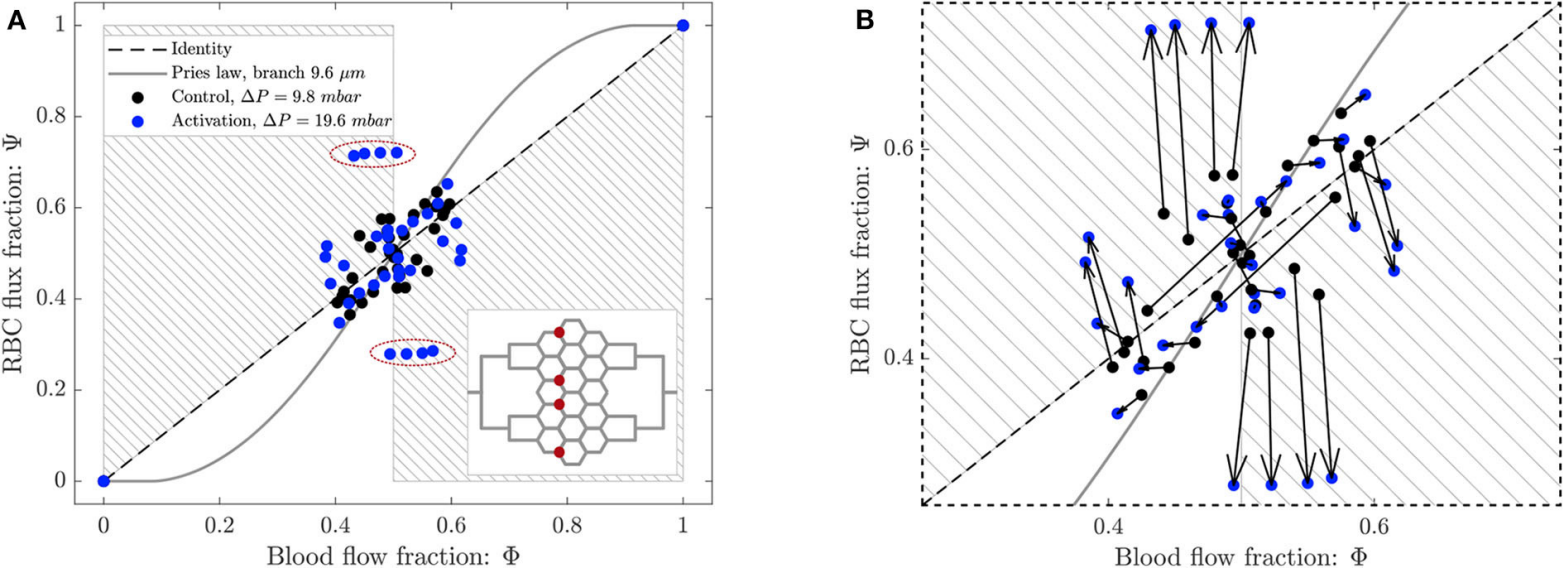
**(A)** Phase separation results for arteriolar activation. Blood flow fraction vs. RBC flux fraction for the control case (black, Δ*P* = 9.8 *mbar*, *p* = 0 *bar*) and the activation of an arteriole (blue, Δ*P* = 19.6 *mbar*, *p* = 0 *bar*). Circles represent data from this study. The gray continuous line is the empirical law from Pries and Secomb (2008). The dashed line is the identity line representing the proportional separation of blood and RBC (no phase separation). Data points in the gray shaded regions indicate an inversion of the Zweifach-Fung effect (Clavica et al., 2016; Shen et al., 2016). In the inset the locations of the four bifurcations with a strong reverse partitioning (circled in red in the Φ − Ψ diagram) are indicated. **(B)** Magnified region of **(A)**. The arrows indicate the change of the phase separation for each bifurcation.

The phase separation results are shown in Figure 10A. The RBC flux fraction Ψ^*i*^ in each daughter branch of all diverging bifurcations was plotted in function of the respective blood flow fraction Φ^*i*^. The results for the control case and the arteriolar activation were compared with the empirical fitting of *in vivo* data from Pries and Secomb (2008).

The data for the control case are the same as in Figure 8 and they are well-described by Pries' separation law. Similarly to the control case, the data for the arteriolar activation lied in the range 0.4 < Φ^*i*^ < 0.6 in the Φ − Ψ diagram. However, the percentage of well-balanced bifurcations decreased to 74% and the deviation from Pries' law appears to be greater. The activation of the arteriole changed the phase separation behavior of many bifurcations. In general, we found that the data points in the Φ − Ψ diagram of Figure 10B had the tendency to depart from Pries' curve toward the regions of reverse partitioning. In fact, many bifurcations presented a more pronounced reverse partitioning compared to the control case. For the diverging bifurcations highlighted in the inset of Figure 10A we observed the strongest reverse partitioning: for 0.5 < Φ^*i*^ < 0.6 we measured a mean $\bar{{\text{Ψ}}^{i}}$ of only 0.28.

A correlation was found between the phase separation results observed for these bifurcations and the local hematocrit distribution in their respective parent vessels. A strong skewness of the hematocrit profile toward one side of the parent vessel (see Supplementary Figure 1D) was probably responsible for the reverse partitioning measured for these bifurcations. This result is consistent with our previous findings (Mantegazza et al., 2020) where the reverse partitioning was found to be more likely for high perfusion pressure and for a skewed RBC distribution in the parent vessel of diverging bifurcations.

## 4. Discussion

In the present study, we designed and fabricated *in vitro* microvascular network models to investigate RBC distribution and phase separation in case of local and global blood flow modulation.

First, to establish the methods and prepare for the experiments with complex network geometries, we investigated the RBC partitioning in a simpler network model (i.e., *single-mesh network*) featuring a side pressure chamber enabling the deformation of a single micro-channel. The increased cross-section due to the inflation of the pressure chamber proved to be an effective mechanism to increase locally the blood flow in one branch of the network and create an asymmetric network configuration.

We found that the degree of asymmetry in the flux partition was positively correlated to the pressure in the chamber (Figure 4). The bigger the deformation in the activated channel, the greater the RBC flux fraction drained into that channel. This is in line with the classical RBC partitioning behavior which follows the Zweifach-Fung effect (Fung, 1973). Moreover, we observed that the asymmetry in the flux partition was negatively correlated to the hematocrit of the suspension. In general, increasing the hematocrit in the blood suspension homogenized the RBC flux distribution at the diverging bifurcation. For example, the RBC flux ratio *q*_*A*/*C*_ at *p* = 2.25 *bar* (Figure 4A) dropped from 1.4 to 1.16 when passing from a 5% to a 25% hematocrit in the blood suspension, thus leading to a more symmetric partition. This is in line with the theoretical considerations by Doyeux et al. (2011) and the results of similar experimental (Fenton et al., 1985; Roman et al., 2016) and numerical studies with idealized (Schmid et al., 2015) and realistic models of capillary networks (Schmid et al., 2019).

We also studied the influence of the inflow velocity on the RBC phase separation when the pressure chamber is inflated. In general, the results are confirming the findings of Clavica et al. (2016) and Mantegazza et al. (2020). Experimental data showed that the asymmetry in the RBC partitioning is negatively correlated to the inflow velocity. Figure 5 shows that a reduction and in some cases even a reversing (data below the NZF limit) of the classical partitioning were observed for increasing perfusion pressure. It has been suggested (Clavica et al., 2016; Shen et al., 2016) that a gradual depletion of the central region of the parent vessel may explain the RBC partitioning measured in our experiments. If the RBCs are marginated, they more likely follow the streamlines of plasma flow and enter into the closer branch, thus reducing the phase separation. Investigating the physical mechanisms at the cellular level that could have led to such hematocrit profiles was beyond the scope of this work. Nonetheless, studies in the literature report that the local hematocrit distribution could be related to the RBC dynamics which is governed by the shear stress experienced by the cells (Lanotte et al., 2016; Minetti et al., 2019) with respect to their deformability. Therefore, the RBC radial migration may be the result of the interaction between lift force, shear rate gradient and non-linear shear induced diffusion (Grandchamp et al., 2013; Losserand et al., 2019). The lateral distribution in the parent vessel of the single-mesh network may also result from the design of the inflow channel which leads to an uneven RBC distribution for higher velocities. For the honeycomb network, we have previously shown in Mantegazza et al. (2020) that uneven RBC distributions are the result of converging streams of RBCs upstream of diverging bifurcations.

Second, we investigated blood flow distribution and RBC partitioning in a more complex microvascular network model (i.e., *honeycomb network*). A local blood flow modulation was achieved by actively dilating a single micro-channel downstream the honeycomb network. This scenario was designed to model the local up-regulation of blood flow induced by a pericyte-mediated capillary dilation. We chose to dilate a channel at the downstream end of the capillary network because the prevailing hypothesis on the vascular spatio-temporal response during hyperemia is that the vessel dilation starts in the cortical capillary network and later propagates upstream toward the arterioles (Hillman, 2014). Conversely, a global increase of the network perfusion was achieved by increasing the perfusion pressure between inlet and outlet of the microdevice. This scenario modeled the blood flow variation induced by the dilation of an arteriole upstream the capillary network. Both of these hyperemic configurations were compared to the same baseline configuration.

Our results showed that the capillary dilation had a significant impact on the global hematocrit distribution (Figure 6A). This is in line with a numerical study investigating blood flow distribution in realistic capillary networks (Schmid et al., 2019) and it may suggest that a localized capillary dilation is not only effective in increasing the flow rate, but also in rerouting the RBCs toward the activated capillary. Moreover, results indicate that a locally confined and heterogeneous RBC velocity variation is only possible by capillary dilation and not by arteriolar dilation (cf. Figures 6B, 9B). As suggested by Schmid et al. (2019), the velocity heterogeneity might be needed for the blood flow redistribution during hyperemia. This hypothesis is supported by *in vivo* experiments reporting positive and negative velocity variations and showing that low and high flux capillaries respond differently during hyperemia (Chaigneau et al., 2003). The very localized nature of the blood flow variation induced by a capillary dilation is shown in Figure 7B. The mean relative difference in the RBC flux is considerably different from the baseline condition only for the first generation of vessel neighboring the capillary dilation (${\bar{Q}}_{rbc}>20\%$). For micro-channels more distant from the pressure chamber the mean relative RBC flux difference remained small ($0\%<{\bar{Q}}_{rbc}<5\%$). The opposite scenario was observed for a global modulation of blood flow. Data indicated that in case of an arteriolar dilation a modulation of blood flow could not be confined to a specific subregion of the honeycomb network. A homogeneous velocity increase was observed throughout the whole network (Figure 9B). At the same time, a variation of hematocrit with a clear global pattern (preferential pathways in Figure 9A) and a more uniform increase of RBC flux (Figure 9C) were observed in preferential regions. This corroborates the hypothesis that a local variation cannot be achieved by dilating relatively big vessels, such as the arterioles. Arterioles are connected with a large number of capillaries, thus any dilation or constriction at the arteriolar level will lead to variation of blood flow in a large region of the microvascular bed.

Phase separation data showed that the diverging bifurcations are generally well-balanced according to the definition given in Schmid et al. (2015). For the baseline as well as for both scenarios reproducing hyperemic conditions, the blood flow fraction was in the range 0.4 < Φ < 0.6 (Figures 8A, 10A). As a result, the average well-balancedness factor was approximately $\bar{B_{db}}\approx 0.8$ for all scenarios that we investigated. This flow characteristic is typical of real capillary networks and it is induced by the RBC dynamics which tends to equalize the outflow velocities, as demonstrated numerically and with *in vivo* experiments by Schmid et al. (2019). It is important to highlight that the well-balancedness persisted even in case of pericyte and arteriolar activation. This indicates that the robustness of the network perfusion is guaranteed at low flow as well as at hyperemia.

Nonetheless, RBCs did not split proportionally to the blood flow fraction in the outflow branches. The majority of the bifurcations followed the classical partitioning governed by the Zweifach-Fung effect and entered the branch with a higher flow. A good agreement was found with the empirical fitting of *in vivo* data proposed by Pries and Secomb (2008). However, at some bifurcations the phase separation behavior was reversed and the RBCs preferred to enter the low-flow branch (gray shaded regions of Figures 8A, 10A). Consistently with our previous findings (Mantegazza et al., 2020), it was possible to identify a strong correlation between the hematocrit distribution in the parent vessels of diverging bifurcations and the type of partitioning. In fact, for all our experiments the bifurcations presenting a hematocrit distribution in the parent vessels very skewed toward one daughter branch (Supplementary Figure 1) were characterized by a reverse partitioning (bifurcations with *S*_*h*_ > 0.1 in Figure 8D). The skewness of the hematocrit distribution was usually enhanced for higher flow velocities (Mantegazza et al., 2020) (Supplementary Figure 1C). These results are a confirmation that the RBC partitioning in complex networks differs from isolated bifurcations. As an example, in Supplementary Figure 1D we report the line density profiles for the bifurcations highlighted in the inset of Figure 10A which had a high skewness factor and presented the most extreme reverse partitioning in the arteriolar activation experiment. As explained earlier, it is likely that the hematocrit skewness is governed by the RBC dynamics and fluid dynamical mechanisms at the scale of RBCs. However, in this case we think that there may be additional contributions due to the network topology, the position of feeding and draining micro-channels and the consequent preferential paths that the RBCs are more likely to follow. For example, bifurcations P1 and P4 in Supplementary Figure 1C are located in the periphery of the network and for each bifurcation there is a daughter branch in which the RBC flow is disadvantaged (Figure 9A). Therefore, the hematocrit distribution in their parent vessels are very skewed toward the center of the network, such that the RBCs can flow through the shortest path connecting the feeding and draining micro-channels.

Our studies suggest that the particulate nature of blood and its heterogeneous partitioning at diverging bifurcations promote the regulation during functional hyperemia. Global and local mechanisms work together to alter the capillary network perfusion. An overall increase of blood supply to the capillary network can be obtained by a dilation of bigger vessels, like the arterioles. Dilations or constrictions at the capillary level are necessary to achieve a fine and very localized blood flow modulation in a specific region of the capillary network. In addition to the well-established role of VSMC at the level of the arterioles, our results may suggest that the activity of the pericytes at the level of capillaries is also relevant in functional hyperemia.

### 4.1. Limitations

The neurovascular coupling is a physiological process involving an ensemble of biological, chemical, electrical and fluid dynamical mechanisms. *In vitro* modeling of all these mechanisms is impossible. A certain degree of simplification has to be accepted when dealing with microfluidic models. For this reason, we did not consider biological or chemical stimuli triggering the neurovascular coupling, but we focused only on its fluid dynamical characteristics.

In the real microvasculature capillaries are cylindrical and their cross-section is not constant over their length. Due to technological limitations, it is not possible to fabricate micro-channels with such geometries in a controlled way at such micro-scale. A first important step in this direction has been made by Fenech et al. (2019), but process controllability and micro-channel reproducibility remains an open issue. In particular, the geometrical differences among different bifurcations are not negligible. This would lead to unpredictable consequences for the RBC flow and partitioning at bifurcations. Considering that bifurcations are our most important test sections, we chose to fabricate straight micro-channels with rectangular cross-section by means of soft-lithography which allows a higher control over the micro-channel geometry.

Our aim was to produce a complex yet idealized micro-channel network inspired by length scales measured *in vivo*. Literature reports capillary diameter between 5 and 10 μ*m* (Wiedeman, 1963; Peppiatt et al., 2006; Peyrounette et al., 2018). Thus, we reproduced the typical confinement that RBCs experience in the capillaries fabricating micro-channels with a characteristic width ≤ 9.6 μ*m*. Some of the findings of this work (i.e., skewness of hematocrit distribution) result from the fact that the channel size is slightly bigger than the RBC diameter. It would not have been possible to observe these phenomena in microchannels with sizes less than or equal to that of RBCs. Results with human RBCs may differ quantitatively due to their larger size compared to porcine RBCs (Reinhart et al., 2017).

Two other differences can be identified between our experimental setup and the perfusion of capillary networks *in vivo*. First, the micro-channel internal walls were coated with a layer of non-specific BSA adsorption while the endothelial surface layer with the glycocalyx (Weinbaum et al., 2007) was not reproduced. Second, our blood mimicking fluid did not contain any blood cellular elements except RBCs. Therefore, it was not possible to investigate the phase separation behavior and RBCs distribution in case of interactions between different blood components.

Despite these limitations, the good agreement between our results and empirical fitting of *in vivo* data (Pries and Secomb, 2008) or numerical data from realistic capillary networks (Balogh and Bagchi, 2018; Schmid et al., 2019) indicated that these simplifications did not prevent the investigation of RBC flow in our artificial networks.

## 5. Conclusions

In the present study, we developed *in vitro* microvascular network models featuring a valve which enabled cross-section variations of a specific micro-channel. This allowed us to investigate the RBC flux distribution and phase separation in models for resting condition and for functional activation. For the first time, we provided quantitative data comparing possible local and global mechanisms for blood flow modulation in a complex *in vitro* network model. Our results are consistent with numerical studies on RBC distribution and phase separation in realistic microvascular networks (Balogh and Bagchi, 2018; Schmid et al., 2019).

Data on RBC phase separation characteristics did not show significant differences between baseline and activation. Classical partitioning (i.e., Zweifach-Fung effect) was the most likely separation mechanism. Reverse partitioning was found for skewed hematocrit profiles in the parent vessel of diverging bifurcation, especially for high flow rates (arteriolar activation).

Our results revealed that a diameter change at the level of a single capillary, as it may be mediated *in vivo* by pericytes, permitted a localized variation of RBC flow and a heterogeneous hematocrit redistribution within the whole micro-channel network (re-distribution of RBCs toward the activated branch). In case of a global increase of the perfusion pressure, as it may be obtained by a dilation of an upstream arteriole, a homogeneous RBC flow increase was found in the whole network and the hematocrit was concentrated toward preferential pathways.

In conclusion, a local and a global modulation of blood flow were tested in a microvascular network and they both proved to be effective in increasing the perfusion of the network. Nonetheless, only a capillary dilation was found to be able to alter the perfusion locally and heterogeneously. It seems reasonable to consider capillary diameter change, possibly mediated by pericytes, as a relevant mechanism for the blood flow regulation during hyperemia.

## Data Availability Statement

The raw data supporting the conclusions of this article will be made available by the authors, without undue reservation.

## Ethics Statement

Blood samples were collected according to the institutional guidelines of the Experimental Surgery Facility (Department of Biomedical Research, University of Bern), and the study protocols were reviewed and approved by the Commission for Animal Experiments of the Office of agriculture and nature of the canton of Bern (animal license BE37/19).

## Author Contributions

AM, FC, and DO designed the microfluidic devices and revised the manuscript. AM conceived and designed the experimental investigations and drafted the first version of the manuscript. AM and MU performed the experiments and analyzed the data. MU performed the fluorescence analysis. DO supervised the scientific work. All authors reviewed the final manuscript.

## Conflict of Interest

The authors declare that the research was conducted in the absence of any commercial or financial relationships that could be construed as a potential conflict of interest.
