# Supplementary material for: Local vs. Global Blood Flow Modulation in Artificial Microvascular Networks: Effects on Red Blood Cell Distribution and Partitioning
Source: Front Physiol. 2020 Sep 25;11:566273. doi: 10.3389/fphys.2020.566273 (PMC7571285; doi:10.3389/fphys.2020.566273)
Supplement: Supplementary file 1 [file Data_Sheet_1.PDF]

Supplementary Material

1 LINE DENSITY PROFILES

A method to compute line density profiles along the width of a micro-channel has been reported by **?**. The region of interest was divided parallel to the axial direction in 20 subregions (each 1 pixel wide) to count the number of RBCs at a given lateral position. Each RBC was assigned to one of this subregions based on its centroid coordinates computed by the particle-tracking software. Line density profiles were computed by dividing the mean RBC number N_{RBC} at each lateral position by the length L_{ROI} of the ROI. The lateral coordinate was then normalized with respect to the width of the ROI: $y^* = y/W_{ROI}$.

The same method has been used here to compute the line density profiles for all diverging bifurcations of the honeycomb network (Fig. 2). Fig. S1 shows the line density profiles for all diverging bifurcations in the honeycomb network for the baseline, for the pericyte and arteriolar activation. It is possible to observe two distinct types of RBC distributions. In fact, the line density profiles were either skewed toward one side of the parent branch of they presented two almost symmetric lateral peaks.

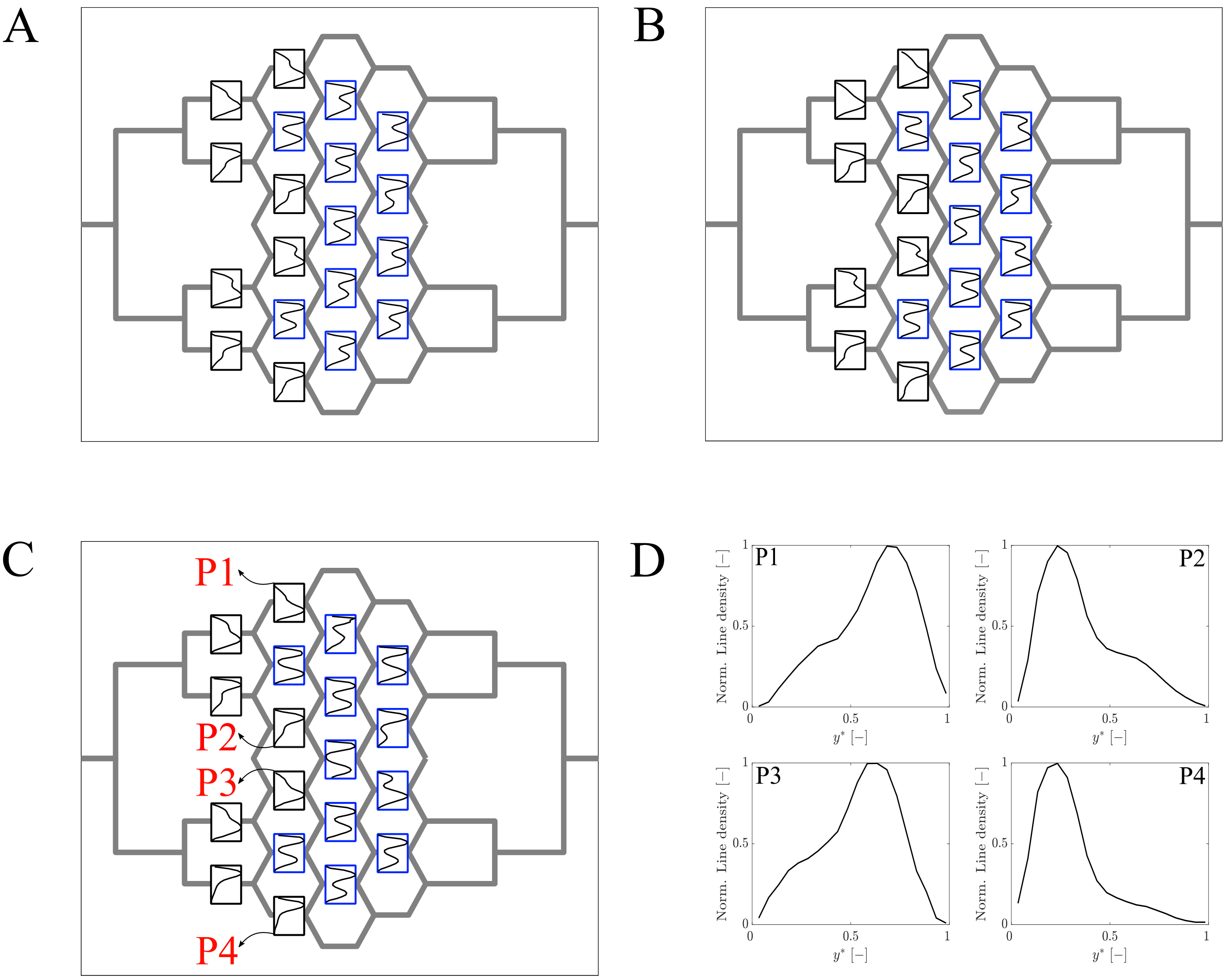

Figure S1. Honeycomb network: normalized line density profiles. Normalized line density for all diverging bifurcations considered in this study for the (**A**) baseline (**B**) pericyte activation and (**C**) arteriolar activation. Line density profiles were grouped based on their shape. (**D**) Samples of normalized line density along the normalized width of the micro-channels ($y^* = y/W_{ROI}$) for the parent vessels of bifurcation P1, P2, P3 and P4.
